# Enteric neuroimmune interactions coordinate intestinal responses in health and disease

**DOI:** 10.1038/s41385-021-00443-1

**Published:** 2021-09-01

**Authors:** Haozhe Wang, Jaime P. P. Foong, Nicola L. Harris, Joel C. Bornstein

**Affiliations:** 1 Department of Immunology and Pathology, Central Clinical School, Monash University, The Alfred Centre, Melbourne, VIC Australia; 2 Department of Anatomy and Physiology, University of Melbourne, Melbourne, VIC Australia

## Abstract

The enteric nervous system (ENS) of the gastrointestinal (GI) tract interacts with the local immune system bidirectionally. Recent publications have demonstrated that such interactions can maintain normal GI functions during homeostasis and contribute to pathological symptoms during infection and inflammation. Infection can also induce long-term changes of the ENS resulting in the development of post-infectious GI disturbances. In this review, we discuss how the ENS can regulate and be regulated by immune responses and how such interactions control whole tissue physiology. We also address the requirements for the proper regeneration of the ENS and restoration of GI function following the resolution of infection.

## Introduction

In mammals, the GI tract contains the largest immune system,^1^ microbiota ecosystem^2^ and endogenous nervous system,^3^ whose intricate interactions help maintain normal GI physiology. In particular, the intrinsic nervous system of the GI tract, known as the ENS regulates vital GI functions.^4^ The disruption of the ENS circuitry can lead to systematic GI symptoms, such as diarrhea, constipation and visceral pain,^4^ which affect up to 40% of the world’s population.^5^

Decades of work have established that substantial communication exists between the ENS and the immune system.^6–11^ Crosstalk between immune cells and the ENS involves the release of soluble mediators such as cytokines, chemokines and neurotransmitters^8–11^ and cognate interactions involving innate immune cells, including mast cells,^12^ macrophages^13,14^, and innate lymphoid cells (ILC).^15–18^ Recent single cell RNA sequencing of human and mouse ENS components revealed that healthy enteric neurons can communicate with both innate and adaptive immune cell types as they express soluble mediators and cell surface molecules that act on immune cells.^19^ The interactions between enteric neurons and immune cells help to maintain the physiological steady state within GI tissues in the absence of pathology, what might be termed tissue homeostasis. Indeed, these interactions may be greater than recognized even 5 years ago and are a dynamic area of research.

Neuroimmune interactions are also important during infection. Altered GI function can promote pathogen clearance via diarrheal responses that can dislodge the pathogen by increasing the local secretion of water into the lumen and promoting propulsive contractile patterns, with both responses requiring input from the ENS.^20–22^ In addition, infection or inflammation-induced disruption of ENS structure and/or activity can promote^23,24^ or reduce inflammation,^13,25^ generate GI symptoms,^26^ and limit tissue pathology.^27^ The ENS can also regenerate allowing for the restoration of GI function after insult.^28–32^ However, complete recovery is often limited in many cases^33,34^ and damage to the ENS can have long-term consequences such as post-infectious GI disorders.^35–37^

This review focuses on the role of enteric neurons in neuroimmune interactions during health and disease with a focus on the complexity of the neural circuits that may underpin these interactions. Our review does not cover all aspects of neuroimmune interactions in detail but aims to integrate information derived from many studies and to highlight advances in knowledge provided by recent publications. We recommend elegant reviews by Yoo and Mazmanian,^8^ Chesné, Cardoso,^9^ Veiga-Fernandes and Mucida,^10^ Verheijden and Boeckxstaens,^11^ Jacobson, Yang,^38^ which expand on the mechanisms underlying neuroimmune interactions in more detail. We will first provide a detailed summary introducing the complexity of the ENS and highlighting some of its key features that are at times overlooked. We then discuss neuroimmune crosstalk in normal physiology, before moving on to discuss the effects produced by infection or inflammatory bowel disease (IBD). Finally, we will examine the key issue of how well the ENS, and its immune system partners, recover after pathological insults.

## The ENS as a semi-autonomous nervous system

**Fig. 1:**
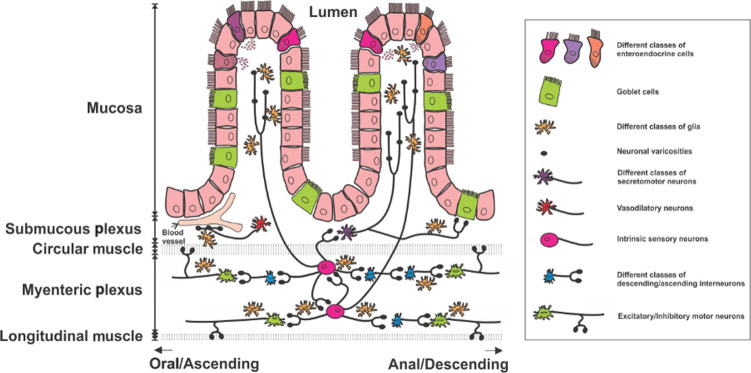
The structure of the ENS. The ENS is made up of neurons and glial cells found within layers of the intestinal wall. Neurons of the ENS are grouped into two main plexi, the myenteric plexus and the submucosal plexus. Intrinsic sensory neurons detect mechanical and chemical signals from the lumen and pass information to secretomotor neurons, vasodilator neurons, interneurons and motor neurons. Enteric neurons communicate via neurotransmitter release from the axonal swelling structures known as varicosities. Several types of epithelial cells line the mucosa layer, including different types of enteroendocrine cells and goblet cells, can also interact with enteric neurons and glial cells to control GI physiology. Foong JPP, Hung LY, Poon S, Savidge TC, Bornstein JC. Early life interaction between the microbiota and the enteric nervous system. American Journal of Physiology-Gastrointestinal and Liver Physiology 2020; 319(5): G541–G548.

The ENS is the largest nervous system (>300 million neurons in humans) outside the brain. It contains all the different types of neurons required to generate complex behaviors including propulsive and mixing contractile patterns, inter-digestive motor patterns and coordination of contractile and secretomotor control (for broad review of historical evidence see Furness^4^). These neurons include intrinsic sensory neurons (ISNs, also known as intrinsic primary afferent neurons or IPANs), interneurons, excitatory and inhibitory motor neurons, secretomotor neurons and vasodilator neurons (Fig. 1). Very recent studies using single cell RNAseq have identified up to 22 distinct classes of enteric neurons based on differing transcriptomes and major regional differences.^19,39–41^ Functions were ascribed to these chemically distinct neurons, but in most cases their anatomical and physiological profiles have not yet been confirmed. Indeed, the RNAseq data underline the extensive immunohistochemical data showing that there are no unique markers able to fully differentiate the functional subtypes of enteric neurons. This complexity complicates interpretation of the studies discussed below, with many of these studies showing that cytokines colocalize with specific enteric neurons classified by only one or two immunohistochemical markers. The cell bodies of enteric neurons are intermingled with very little functional segregation in clusters (ganglia) of 2 to >300 neurons connected by nerve trunks that form both connections between ganglia and to nerve plexi that innervate the mucosal epithelium, the crypts and the intestinal smooth muscle. There are two main ganglionated plexi known as the myenteric plexus, in which the ganglia are larger, and the submucosal plexus. The myenteric plexus contains most of the neurons that regulate intestinal contractile patterns and the circuits that coordinate these patterns with other intestinal behaviors, while the less well studied submucosal plexus primarily contains neurons that project to the mucosa and are implicated in regulation of secretion, vasodilatation and absorption. Some myenteric interneurons and ISNs innervate the submucosal ganglia^42,43^ and conversely, some submucosal neurons project to the myenteric plexus (Fig. 1).^44–46^ The architecture of the ganglia and the neurochemical profiles of enteric neurons differ significantly between intestinal regions reflecting the functional differences between the different parts of the small intestine and the proximal and distal parts of the colon.^47,48^ Therefore, caution is required when attributing function identified at one region to another without further investigations.

The enteric neural circuits that regulate gastrointestinal (GI) behaviors are modulated by inputs from the parasympathetic nervous system via the vagus and sacral part of the spinal cord and by inputs from the sympathetic nervous system. These extrinsic inputs serve to integrate enteric network activity with the requirement of the whole organism during homeostasis, physiological, or psychological stress and during pathological conditions. They are informed by a variety of extrinsic primary afferent neurons with cell bodies in the nodose or dorsal root ganglia and whose peripheral terminals innervate the different layers of the gut to sample the local environment (for review see Furness^49^).

Enteric circuits also receive input from, and probably provide output to, enteroendocrine (EE) cells that synthesize and secrete many different hormones and other mediators in the mucosa (Fig. 1).^50^ This is most prominent for a subclass of EE cell, enterochromaffin (EC) cells, which synthesize and release 5-hydroxytryptamine (5-HT, serotonin).^51,52^ EC cells are mechanosensitive but also respond to various chemical stimuli in the mucosa to release 5-HT onto the mucosal terminals of some enteric (and extrinsic primary afferent) neurons.^53,54^ Although motor activity is seen in the absence of EC 5-HT,^55^ there is strong evidence that mucosal 5-HT release modifies intestinal motor patterns or even initiates them^56–59^ and that this release can be triggered by smooth muscle contractions.^60^ Release of 5-HT from EC cells also activates secretomotor pathways leading to electrogenic secretion of water and salt into the lumen.^61^ Thus, EC cells and probably EE cells should be considered integral parts of the enteric circuit.

Enteric neurons synthesize and release a wide range of neurotransmitters and neuropeptides, allowing them to communicate with smooth muscles, epithelium, other neurons and possibly immune cells. These target cells are all located 100 μm to >10 cm away from the neuron’s cell body (for review see Furness^49^). The release of neurotransmitter requires the generation of action potentials in their axons, allowing signals to reach cells that are distant. In neurons, small molecule neurotransmitters like the major excitatory enteric transmitter acetylcholine (ACh) are synthesized within or taken up into the nerve terminals. In contrast, neuropeptides that may act as neurotransmitters are synthesized as precursor proteins and processed to the mature form during the long journey from the Golgi apparatus in the cell body to the terminals. This means that cell bodies of enteric neurons often have only low levels of their characteristic neuropeptides, which are not detected unless fast axoplasmic transport is blocked.^62^ Nevertheless, most enteric neurons have several putative neurotransmitters in their nerve terminals together with a range of other neuroactive mediators. These can include ACh and potentially excitatory neurotransmitters like ATP, 5- HT, glutamate and excitatory neuropeptides like substance P (SP) and vasoactive intestinal peptide (VIP) together with inhibitory peptides like enkephalin and dynorphin.^63^ Several of these have different functions depending on the target cells and their receptors. For example, 5-HT can be excitatory via some of its receptor subtypes and inhibitory via others and is only found in interneurons,^52,63^ while both ATP and VIP excite neurons, but inhibit the smooth muscle when released from inhibitory motor neurons.^64^ The primary inhibitory transmitters to the smooth muscle are nitric oxide (NO), synthesized on demand by neuronal nitric oxide synthase (nNOS, NOS1), and ATP, but many inhibitory motor neurons also contain VIP.^64,65^ VIP is the primary transmitter of most submucosal secretomotor neurons and is a potent secretagogue on newly differentiated epithelial cells,^66^ but some VIP containing submucosal neurons express markers for other neurotransmitters like nNOS and tyrosine hydroxylase.^48^ Importantly, the exact cellular targets of these subtypes are yet to be identified, and answering such questions can help us better understand the full functions of submucosal neurons. ACh is the primary secretagogue released by the other secretomotor neurons but may act as a vasodilator transmitter on submucosal arterioles.^66^ Amongst other mediators, the cholinergic secretomotor neurons also express calcitonin gene related peptide (CGRP),^48,67^ which is often assumed to label both extrinsic primary afferent neurons and myenteric intrinsic sensory neurons.^68^

Importantly, where neurotransmitters are stored and released is critical for their function and how they can interact with immune cells. Typically, enteric axons exhibit swellings, varicosities, at many sites along their length and most, if not all, of these have the features of nerve terminals with clusters of transmitter containing vesicles and what appears to be the normal release apparatus (Fig. 1).^4^ This has led to the conclusion that varicosities are the enteric equivalent of central synapses, but many do not form close contacts with other neurons or the muscle and those that do frequently do not align with a postsynaptic specialization.^69^ Detailed analysis of varicosities of other autonomic axons has shown that the probability of release from these structures can be very low during a single action potential.^70^ This suggests that mediator release at neuronal varicosities likely requires long high frequency trains of action potentials, commonly referred to as ‘bursts’ of action potentials. Furthermore, a mediator released by a cell such as an immune cell or a glia cell contacting a neuronal axon can only really affect the neuronal cell body or other axonal varicosities (which may be over a centimeter distant) by generating an action potential within the neuron, or via anterograde or retrograde axoplasmic transport of a signaling molecule (this would be expected to be many orders of magnitude slower than an action potential). Addressing these issues in relation to the intimate contacts between enteric neuronal axons and immune cells should substantially enhance our understanding of neuroimmune interactions.

It is generally believed that, in the absence of pathology, enteric neurons are long-lived and maintained throughout adult life^71,72^ but recent controversial evidence suggests adult enteric neurons turn over rapidly indicating that the ENS may be more dynamic than previously thought.^73^ Importantly, it is clear that enteric neurons undergo cell death following pathological insult, while damaged axons of surviving neurons regenerate.^28–32^ For detailed review of ENS structure and functions, refer to Furness,^49^ Spencer and Hu.^74^

Enteric neurons are surrounded and supported by another major cell population, enteric glial cells. These are found throughout the axons within or between ganglia, in the muscle and lamina propria and in close relationships with the mucosal epithelium. They provide structural and metabolic support for neurons and their axons,^72^ modulate synaptic signaling,^75^ contribute to regulation of motility patterns^76^, and help maintain intestinal barrier integrity.^77,78^ Intriguingly enteric glia are connected via gap junctions^79^ and hence can communicate with each other metabolically and via changes in intracellular Ca^2+^ levels. They also receive input from enteric neurons through purinergic (ATP) and other signaling pathways,^80–82^ and can activate enteric motor patterns via similar mechanisms.^75,83^ Importantly, enteric glia can differentiate into enteric neurons following chemically induced tissue injury,^29^ inflammation, or infection.^84^ Thus, there is two way communication between enteric neurons and glia both in signaling and in recovery from pathological insults. Enteric glia also mediate neuroimmune interactions, and please refer to Chow and Gulbransen,^85^ Veiga-Fernandes and Pachnis^86,87^ for more detailed reviews.

## Neuroimmune crosstalk during homeostasis

The mucosal immune system needs to tolerate microbiota symbionts^88^ and yet recognize, limit and eradicate diverse disease-causing pathogens.^89,90^ This section focuses on how the ENS participates in the regulation of mucosal immunity with important consequences for tissue homeostasis. For discussion of the roles of extrinsic nerves in the neuroimmune interactions in the GI tract see recent reviews by Verheijden and Boeckxstaens,^11^ Margolis, Gershon.^91^

## ENS modifies mucosal immunity

**Fig. 2:**
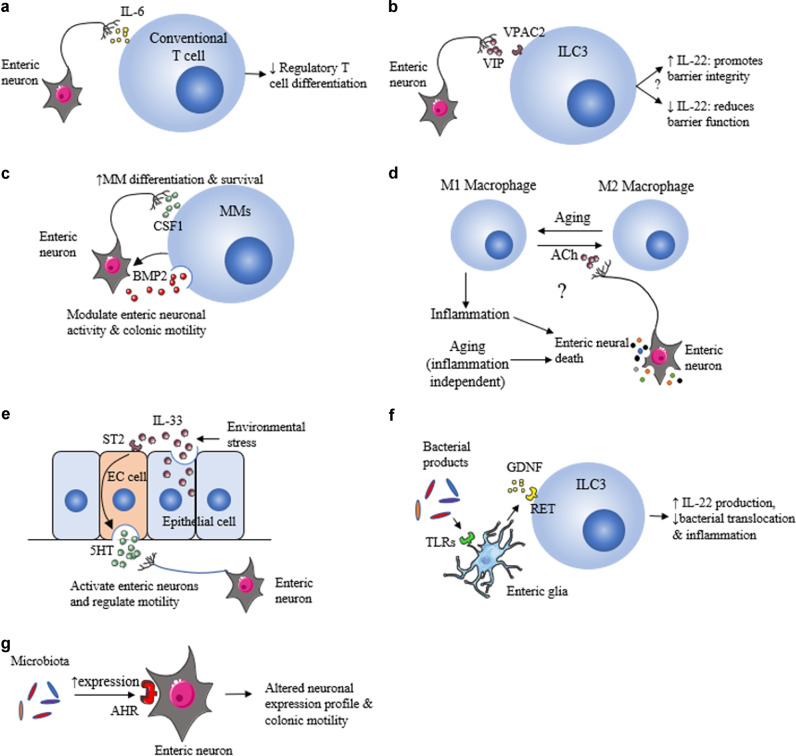
Enteric neuroimmune interactions during homeostasis. **a** IL-6 derived from enteric neurons reduces regulatory T cell differentiation. **b** VIP secreted by enteric neurons can bind to VIP receptor type 2 (VPAC2) expressed by ILC3s. VIP promotes or suppress IL-22 release from ILC3s, resulting in increased or decreased barrier function, respectively. **c** Enteric neurons secrete CSF1 to promote the survival and differentiation of muscularis macrophages (MM). In turn, MMs release bone morphogenic protein (BMP) 2 that excites enteric neurons and regulates colonic motility. **d** Hypothetical interactions between MM and enteric neurons during aging. Aging promotes phenotypical switches of MM from M2 to M1, leading to intestinal inflammation and consequent neuronal death. Aging itself can also cause neuronal loss, especially for cholinergic neurons. This may exacerbate inflammation as ACh can promote MM phenotypic switch to M2. **e** Environmental stress results in epithelial cells release of IL-33, which bind to ST2 receptors on enterochromaffin (EC) cells and promotes the release of 5-HT. Increased 5-HT modulates neuronal activities and increases intestinal motility. **f** Bacterial products bind TLRs on enteric glia leading to secretion of GDNF, which then binds to RET receptors on ILC3’s to promote IL-22 production. This limits bacterial translocation across the epithelium and reduces inflammation. **g** The microbiota promotes expression of AHR on enteric neurons, which in turn regulates the neuronal transcriptional profile and changes colonic motility. Creative Commons Attribution 3.0 Unported (CC BY 3.0).

Both myenteric and submucosal neurons express a variety of immune mediators including Interleukin (IL)-6 and IL-18.^27,92^ Gene transcripts for some cytokine receptors, such as IL-4 receptor alpha (rα) rα and receptors for TNF and TGF-β are expressed ubiquitously by ENS neurons—whilst the genes for IL-10rβ, IL-11rα1, IL-13rα1, IL-17r, and IL-20rα, are selectively expressed by subsets of enteric neurons.^19^ Functionally, enteric neuronal IL-6 is reported to suppress regulatory T cell differentiation during homeostasis (Fig. 2a).^92^ Further, many neurotransmitters co-localized in these neurons and their terminals also mediate immune functions. SP has pro-inflammatory functions and supports the migration, proliferation, and activation of multiple immune cell types.^14,23^ By contrast, VIP has anti-inflammatory functions and can inhibit macrophage and neutrophil activation, reduce pro-inflammatory cytokine production and promote regulatory T cell and T helper 2 (Th2) cell differentation.^25^ VIP has also been reported to trigger^15^ or inhibit^93^ IL-22 production from type 3 ILC3 (ILC3), which in turn contributes to intestinal barrier integrity (Fig. 2b). These apparently contradictory roles of VIP may arise because there are a minimum of six different classes of VIP neurons in the ENS including myenteric interneurons containing NOS, other interneurons that are cholinergic, inhibitory motor neurons and three types of submucosal VIP neurons distinguished by the presence of NOS and other markers.^48,62,94–96^ Thus VIP release may play different roles, depending on what neuronal subset it is being released from. Nevertheless, the possibility that ILC3 activity and IL-22 production can be modulated by variations in VIP levels during the circadian cycle reveals how this cycle influences neuroimmune interactions in the GI tract. Identifying the specific neurons that alter their VIP levels by feeding during the circadian cycle is essential to understand how such interactions influence the ENS circuits. Moreover, as terminals of these VIP axons may also contain ACh or NO, it is important to investigate whether ACh or NO affect ILC3 activation.

ACh also has anti-inflammatory functions and can limit pro-inflammatory cytokine release via its ability to activate the α-7 nicotinic receptor (α7nAChR) expressed on various immune cells such as macrophages.^97,98^ Moreover, 5-HT may also participate in neuroimmune interactions as many immune cell types express 5-HT receptors or other proteins in 5-HT pathways.^99^ As such, 5-HT contributes to diverse immunomodulatory functions including the recruitment of neutrophils, the activation of T cells and reduction of IL-1β and TNFα production.^99^ Glial cells can also secrete immune mediators, with the key glial cell molecule, S100β, reported to promote the production of pro-inflammatory cytokines by macrophages.^100^

Whilst it is evident that the ENS can modulate mucosal immunity, further work is necessary to fully characterize the impact of ENS secreted molecules on immune cells and to investigate how this may differ in the fed versus the fasted state. For example, the ENS generates a “migrating motor complex” in the fasted state, which starts in the distal antrum then propagates to the ileo-caecal junction and serves to “clean” the intestinal lumen of mucosal debris and gut microbes. This migrating motor complex is represented by a period of intense neural activities involving activation of motor neurons that lasts for at least 5 min at any spot along the small intestine and is repeated every 90–120 min in humans.^101,102^ A key question here is how cytokines are released from neurons. Are cytokines, like neurotransmitters, contained within vesicles from varicosities with their release occurring as the result of a single and/or a burst of action potentials? If so, cytokine release, or even the overall GI immune activities, may be synchronized with neuronal activities and therefore become rhythmic.

## Muscularis macrophages interact with the ENS

Perhaps the most well documented neuroimmune interactions are those between muscularis macrophages (MMs) and enteric neurons and glial cells in the myenteric plexus.^13,14,103^ During homeostasis enteric neurons support the survival and differentiation of MM by secreting colony stimulatory factor 1 (CSF1). In turn, MM secrete bone morphogenic protein 2 (BMP2) that activates enteric neurons expressing BMP2 receptors to regulate colonic peristalsis (Fig. 2c).^14^ Interestingly, MM in the postoperative ileum respond to ACh released from ChAT + myenteric neurons by producing fewer pro-inflammatory cytokines^13^ and promoting the restoration of impaired gastric emptying that occurs after surgery.^104^ MMs may also contribute to ENS homeostasis by phagocytosing apoptotic neurons, thereby supporting neuronal turnover.^73^ Notably, aging can shift intestinal macrophages from an anti-inflammatory M2 phenotype to the pro-inflammatory M1 phenotype, leading to intestinal inflammation. Such age-dependent macrophage phenotypical changes are associated with reduced numbers of neurons and neural stem cells, increased neuronal apoptosis and increased intestinal transit time.^105^ In addition, aging associated enteric neuronal loss may also be inflammation independent.^106^ An intriguing aspect of this is that neurons releasing ACh are reported to be specifically vulnerable to age related loss^106^ and ACh, acting via α7nAChR, can convert M1 macrophages to the M2 phenotype (Fig. 2d).^107^ Thus, it is important to know if there is any specificity of the close interactions between MMs and the types of enteric axons they surround.

## The involvement of enteroendocrine cells

Physiological neuroimmune interactions can also take place indirectly via EC cells, with a recent report demonstrating that IL-33 release during GI homeostasis triggers release of 5-HT from EC cells and thereby altering intestinal motility (Fig. 2e),^108^ and this is likely to also promote secretion.^109^ To date, the signals underlying IL-33 release during intestinal homeostasis remain unclear, with one possibility being focal cellular necrosis.^110^ Some EE cells have basal processes that synapse with axons,^50^ raising the possibility that sensory roles of EE cells are directly integrated into the neuroimmune circuit.^111^ Given the important signaling roles of EE cells, such possibilities are worthy of investigation.

## The intestinal microbiota

The intestinal microbiota is essential for maintaining homeostasis, with dysbiosis leading to impaired ENS development including reduced neuronal densities, altered neuronal subtypes, and changed electrophysiological function. Together, with the known impacts of the microbiota on immune cell function, such alterations can result in impaired ENS function and reduced gut motility.^112–117^ The microbiota is also essential for the postnatal development and maintenance of glial cells.^118^ Cells of the ENS can directly or indirectly sense and respond to microbial and immunological cues. Both enteric neurons and glial cells express pattern recognition receptors including toll-like receptor (TLR)-2, TLR-3, TLR-4 and TLR-7.^119–121^ Interestingly, the activation of TLR2 and TLR4 on glial cells by commensal bacterial products can increase the release of glial cell line-derived neurotrophic factor (GDNF)-family ligands that bind to RET expressed on ILC3 (Fig. 2f), stimulating the secretion of IL-22 and providing protection against chemical-induced inflammation or pathogenic bacterial infection.^18^

Notably, the presence of microbiota in the colon of mice promotes the expression of aryl hydrocarbon receptor (AHR) by myenteric neurons, allowing these neurons to sense the luminal environment (Fig. 2g).^115^ The downstream signaling of AHR regulates the myenteric neurons’ activity, which in turn affects colonic motility.^115^ Importantly, re-colonization of germ-free mice can restore proper ENS function and intestinal physiology, at least in part by impacting the serotonergic system.^122,123^ The ENS has a reciprocal impact on the microbiota, with genetic ablation of ENS development impacting microbial communities in both humans and zebrafish, and even subtle changes in expression of synaptic proteins affecting microbial communities in mice.^124–126^

In addition, recent studies reveal interactions between gut microbiota and Hydra prototypical pacemaker neurons, cells that resemble intestinal smooth muscle pacemakers in mammals. In the absence of microbiota, Hydra neurons downregulate their pacemaker gene,^127^ thereby reducing Hydra body contractions.^128^ Recolonization of microbiota largely restores pacemaker gene expression and improves motility.^127,128^ Hydra neurons are enriched with immune-related genes, and secrete antimicrobial proteins (AMP).^127,129^ These results support the hypothesis that the nervous system and the immune system work synergistically through evolution, and may even share a common ancestral cell.^86,91^

## Pathogenic infection and ENS function

Neuroimmune interactions are also apparent following GI infection and function to expel pathogens residing in the lumen. Here we focus on studies that reveal involvement of the ENS in neuroimmune interactions during pathogen infection.

## Pathogenic bacterial infection

Diarrhea is the most important pathological symptom of infection with *Vibrio cholerae* whose exotoxin, cholera toxin (CT), can directly target epithelial cells to promote secretion.^130^ The ENS contributes to this process, as CT elicits hyperexcitability of secretomotor neurons and ISNs resulting in hyperactive enteric secretomotor circuits.^26,131^ Interestingly, some enteric neuronal subtypes are more vulnerable to CT than others. For example, only cholinergic secretomotor neurons are hyperexcitable in mouse submucosal plexus after CT exposure.^132^ However, CT increases the excitability of guinea pig submucosal non-cholinergic VIP neurons,^26^ and VIP plays a major role in CT induced hypersecretion in rats,^133^ which suggests significant species differences. Moreover, the effects of CT on the gut can be sexually dimorphic and sensitive to hormone levels.^59^

Toxins from *Clostridium difficile* also impact the ENS. Application of toxin A directly onto submucosal plexus of guinea pigs excites ISNs and suppresses inhibitory synaptic transmission from the sympathetic nerves that innervate this plexus.^134^ Toxin A also excites rat extrinsic neurons, causing the release of SP from extrinsic afferent neurons that excites neurons in the myenteric and submucosal plexi.^135^ By contrast toxin B activates IL-1β production by monocytes^136^ resulting in the activation of VIP + secretomotor neurons,^137^ thereby promoting fluid secretion.^66^ Toxin B also increases neuronal IL-8 production.^138^

**Fig. 3:**
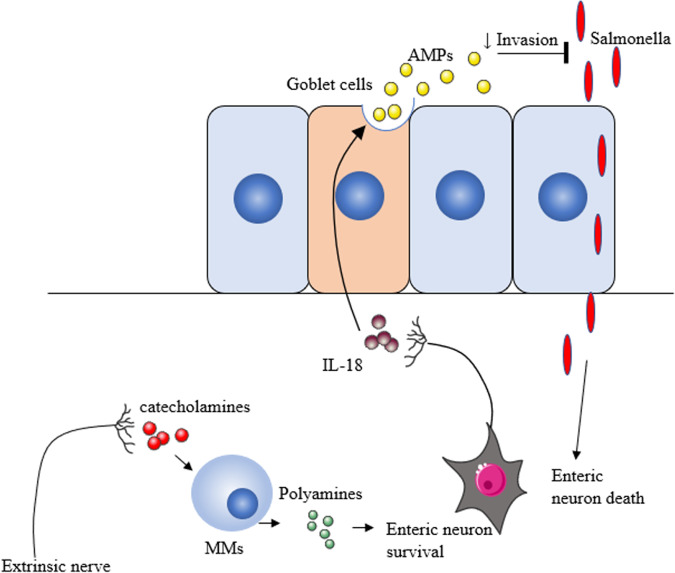
Neuroimmune interactions in salmonella infection. Invasion of salmonella causes the death of enteric neurons. However, catecholamines produced by extrinsic nerves induce MM to secrete neuronal protective polyamines, which promotes the survival of enteric neurons. Enteric neurons also release IL-18 which stimulates the production of antimicrobial proteins (AMPs) by goblet cells and reduces invasion of salmonella across the epithelial barrier. Creative Commons Attribution 3.0 Unported (CC BY 3.0).

Bacterial infections can also lead to neuronal loss. Mice infected with an attenuated strain of *Salmonella typhimurium* exhibit a prolonged loss of myenteric neurons including a subset of excitatory neurons identified by a decrease in the number of vesicular glutamate transporter 2 positive (vGlutT2) neurons.^33^ Unfortunately, this study did not examine survival/loss of ACh + neurons, which are the major excitatory subtype in the myenteric plexus and represent 70% of all neurons. Of note neuronal loss was prevented by genetic ablation of Nlrp6 (NOD-like receptor family pyrin domain containing 6) and Caspase 11 within neurons, indicating it occurred as a result of inflammation. Interestingly, vGluT2 neurons express higher levels of Nlrp6 mRNA, corresponding with their preferential vulnerability.^33^ In addition, catecholamines released by extrinsic nerves in response to infection activated β2 adrenergic receptors on MMs, resulting in an increased expression of Arginase 1 and production of neuroprotective polyamines (Fig. 3).^33^ Thus, infection-induced alterations to the ENS or extrinsic nerves can be both detrimental and protective for the host.

The importance of neuroimmune interactions in response to bacterial infection is further highlighted by the discovery that neurons are a key source of protective IL-18 in response to Salmonella infection.^27^ During such infections, enteric neurons release IL-18 to promote AMP secretion from goblet cells via a caspase 1-independent mechanism, leading to improved pathogen control (Fig. 3).^27^ This highlights the ENS as an important component of the host protective response and raises the question of why neurons, rather than IL-18 competent cells were key to protection. One **possible** answer could be the ability of the ENS to rapidly transmit signals between different regions (the conduction velocity of enteric axons is ~50 cm/s^139^ allowing neuronal activities to travel a distance of 1 m within 2 s) to enable fast host responses against the infection. The ENS may also target bacteria directly as various neuropeptides such as SP, VIP, CGRP and neuropeptide Y (NPY) can reduce bacterial growth in vitro.^140^ Strikingly, VIP can kill bacteria directly by disrupting bacterial membrane integrity.^141^ Investigating the potential bactericidal activity of neuropeptides may be a novel avenue for anti-microbial drug discovery.

## Parasitic infections

Intestinal helminth infection has long been known to trigger type 2 immune responses, characterized by the release of IL-4, IL-5, and IL-13 from type 2 ILC (ILC2) to CD4 + T cells (Th2), that function to accelerate parasite expulsion^90,142,143^ via the ‘weep and sweep’ response.^144^ In keeping with this, helminth infections are also accompanied by alterations to the ENS.^145^

### Helminths change the ENS

Helminth infection alters enteric neuron morphology, electrophysiology, and neurochemistry. These changes are highlighted in a study of *Trichenalla spiralis* infection in guinea pigs where jejunal ISNs exhibited heightened neuronal excitability and synaptic plasticity.^146^ Such changes probably contribute to the commonly observed alterations in intestinal physiology that include increased secretions and motility. Indeed, hypersecretion of fluids into rat intestinal lumen in response to *Nippostrongylus brasiliensis* infection is largely alleviated by inhibition of nicotinic ACh receptors or voltage gated sodium channels to block neuronal action potentials, indicating neuronal involvement.^20^ The increased smooth muscle contractions characteristic of *N.brasiliensis* and *Heligmosomoides polygyrus* infection in mice are also, at least in part, driven by neuronal alterations.^147^ In addition, *Hymenolepis diminuta* induces myoelectrical changes in rat small intestine, a phenomenon that is reduced by intestinal transection which damages the integrity of the ENS.^148^

Helminths also alter the availability of neuropeptides with increased SP and/or reduced VIP reported following infection with *N. brasiliensis*, *T. spiralis, H. diminuta*, *Echinostoma liei*, and *Schistosoma japonicum*.^24,149,150^ Further, ACh release is reduced in myenteric plexus of rats infected with *T. spiralis*.^151^ However, it should be noted that altered availability of other ENS neurotransmitters varies across studies, likely due to the use of different helminth and/or host species.^149^ Nevertheless increased SP is of particular interest as SP promotes recruitment of neutrophils and lymphocytes and activates a vast array of immune cells, including natural killer cells, macrophages, dendritic cells, neutrophils, mast cells, eosinophils and T cells.^23,24^ Its importance in helminth infection is supported the finding that deficiency of SP or its receptor, neurokinin 1, results in impaired cytokine production following infection with *Taenia crassiceps*.^152^ In addition to its well-known role in the activation of host type 2 immunity following helminth infection,^110^ a recent report revealed that IL-33 also promotes the expulsion of *T. muris* from mice by eliciting the release of 5-HT from EC cells causing increased intestinal motility.^108^ 5-HT is known to enhance neurogenic fluid secretion,^109^ which may also contribute to parasite clearance.

### Neuro-ILC2 interactions during helminth infection

**Fig. 4:**
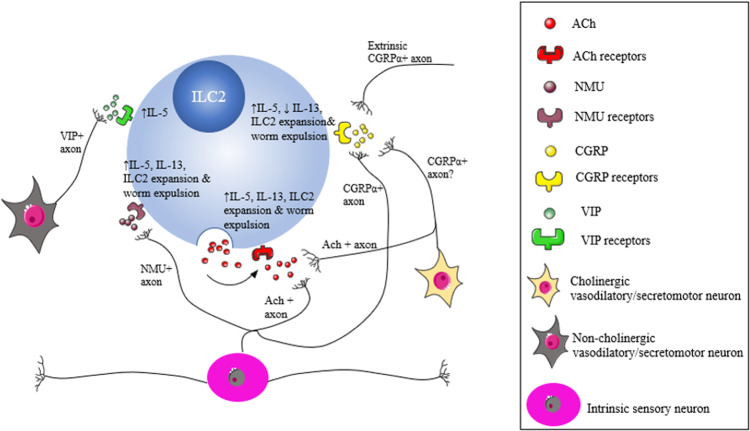
Possible network of neuroimmune interactions between enteric neurons and ILC2. In brief, ISNs (which originate in the myenteric plexus but have multiple long processes allowing them to contact cells in the lamina propria) represent the sole source of NMU in the GI tract and can also secrete CGRPα and ACh. Of note, ISNs also express receptors for IL-4 and IL-13, and a subset of these neurons express the NMU receptor, Nmur2. Thus, ISNs have the potential to participate in bi-directional communication with ILC2s during homeostasis and following helminth infection. In the submucosal plexus cholinergic vasodilator/secretomotor neurons secrete ACh and potentially CGRPα and therefore may also interact with ILC2s, whilst non-cholinergic vasodilator/secretomotor neurons may interact with homeostatic ILC2s via their secretion of VIP. Extrinsic primary afferent neurons may also regulate ILC2 activities via CGRPα. Creative Commons Attribution 3.0 Unported (CC BY 3.0).

Recent work has highlighted intimate interactions between enteric neurons and ILC2 within the lamina propria of helminth infected mice (Fig. 4). ILC2s constantly secrete IL-5 in the absence of pathogens,^153^ whereas *N.brasiliensis* infection results in an increase in their number^154^ and secretion of IL-5 and IL-13. Both an increase in ILC2s and Th2-like mediators play an essential role in the expulsion of the parasite.^90^ This may be mediated by release of Neuromedin U (NMU) from cholinergic neurons in response to parasite products, elicited ILC2 proliferation and increased IL-5 and IL-13 production.^16,155^ These neurons are likely to be ISNs, because NMU appears to be confined to some subtypes of this functional class of enteric neurons.^19,41,156^ Another report highlighted intimate contacts between ILC2s and CGRPα + neurons,^154^ with these neurons functioning to suppress ILC2 proliferation and IL-13 production, resulting in impaired worm expulsion.^17,154^ This CGRPα could be released by several types of neurons, including ISNs,^19,154^ extrinsic primary afferent neurons,^157^ and probably also some cholinergic secretomotor neurons.^48^ Interestingly, although IL-13 production was suppressed, CGRP promoted IL-5 production by ILC2s.^158,159^ VIP can also promote IL-5 secretion from ILC2 in response to feeding,^153^ but this effect may be diminished **in** the pathological state because as helminth infection has been reported to decrease VIP.^149,150^ Lastly, two very recent publications demonstrated that ILC2s express mRNAs for both muscarinic and nicotinic receptors and secrete ACh in response to IL-33 and *N.brasiliensis* infection.^158,159^ ACh synthesis acted in an autocrine manner to promote IL-13 and IL-5 production, together with proliferation, of ILC2s and contributed to parasite expulsion.^158,159^ The increased expression of cholinergic receptors would also make these ILCs more sensitive to their neural inputs, which include ISNs and cholinergic secretomotor neurons as discussed earlier. Taken together these reports indicate that mucosal ILC2s interact with terminals of several classes of enteric neurons. Although still hypothetical, Fig. 4 summarizes the potential interactions between ILC2s and different subsets of enteric neurons.

### Other immune cells in helminth infections

Besides ILC2, other immune cells associated with helminth infection, notably mast cells, alternatively activated macrophages and basophils, may also mediate neuroimmune interactions. Around 70% of enteric mucosal mast cells make intimate contacts with the ENS,^12^ and neurally released mediators such as ATP and the neuropeptides, SP and CGRP, can induce mast cell degranulation, whilst somatostatin suppresses mast cell activation.^12^ Activation of human mast cells by enteric neurons has been visualized by calcium imaging experiments in which electric stimulation of these neurons triggered activation of adjacent mast cells. This phenomenon is abolished by blocking nerve action potentials and reduced by blocking CGRP and VIP receptors—highlighting these as likely neurotransmitters mediating mast cell activation.^160^ Although the same approach revealed only rare events of neuronal excitation following mast cell activation in macroscopically healthy tissue,^160^ histamine directly excites enteric neurons and generates patterned neuronal activity.^12,161^ It is possible that mast cell-neuron communication occurs more frequently in diseased intestine, such as during helminth infection during which mast cell degranulation events are increased. Macrophages activated by type 2 cytokines to adopt an alternative phenotype are well known to control inflammation, repair tissue damage and to trap tissue migrating helminth larvae during helminth infection.^162^ Thus, it is possible that the ENS may assist the tissue protective roles of macrophages in response to helminth infection.

Although it is currently unknown whether basophils interact with enteric neurons, basophils are reported to change morphology and make contact with skin sensory nerves during atopic dermatitis, with the basophil-derived leukotriene C4 functioning to activate dorsal root ganglion neurons and promote acute itchy flare.^163^ Thus, it is possible that basophils can also modulate ENS functions during helminth infection, and this may be a promising avenue for future research.

Despite the recent surge in reports detailing neuroimmune interactions following GI infection, much remains unknown. For example, in natural settings helminths often remain in their host for years, raising the question as to whether the ENS actively participates in such tolerance mechanisms. As food allergy produces similar type 2 immune responses to helminth infections,^164^ it is likely that the neuroimmune interactions mentioned above also participate in allergic responses. Moreover, the body’s response to infections, such as the ‘weep and sweep’ response, can alter intestinal bacterial communities and the resulting dysbiosis may contribute to disease pathology and/or long-term alterations in GI function, as discussed in later sections. Addressing such questions will likely prove important for our understanding of both ENS and immune function within the GI tract and may potentially assist the design **of** new therapeutics for GI symptoms.

## Viral infection

Viral infections also impact enteric neurons. Rotavirus induces diarrhoea by stimulating the release of 5-HT from EC cells to activate secretomotor pathways and trigger propulsive intestinal motility.^165,166^ West Nile virus (WNV), a neurotrophic flavivirus, can infect myenteric and submucosal neurons in mice leading to altered GI motitliy^167^ A recent report investigating the impact of GI helminth infection on the host response to WNV showed that co-infection markedly increased disease pathology by eliciting a type 2 immune response leading to increased bacterial translocation across the intestinal epithelium, impairing the WNV-specific immune response and increasing viral titers.^168^ Co-infection also rendered enteric neurons more susceptible to the virus and resulted in a loss of calretinin + and nNOS + myenteric neurons.^168^ Thus, how GI infections change the ENS may depend on the pathogen and the nature of the immune response it elicits. Although difficult to investigate, the prevalence of GI co-infections provides ample incentive for the further investigation of neuroimmune interactions in such complex settings.

## Inflammation and the ENS: focus on inflammatory bowel disease

Communication between the ENS and immune system also contributes to non-infectious GI disorders, notably IBD. IBD involves changes in both immune and ENS function, with potential alterations to neuroimmune interactions as detailed below.

## Inflammatory bowel disease

IBD is a broad term encompassing both ulcerative colitis and Crohn’s disease. Crohn’s disease commonly affects the distal small intestine and colon and is typified by a type 1 or type 17 immune response with increased production of IL-12, IL-17, IL-23, IFN-γ, and TGF-β. In contrast, ulcerative colitis is restricted to the colon and is more often characterized by a type 2 response, including higher levels of IL-5 and IL-13.^169–171^ Such inflammatory environments can directly alter ENS functions. Two cytokines usually ascribed a pro-inflammatory function, IL-1β and IL-6, directly depolarize guinea pig enteric neurons and elicit action potential firing, an effect suppressed by IL-1 receptor antagonist.^172^ Interestingly, IL-1β and IL-6 appear to suppress both excitatory and inhibitory synaptic transmission.^172^ Glial cells in mice were also recently demonstrated to contribute to intestinal inflammation via the release of soluble macrophage CSF via connexin-43 channels to activate MMs and polarize them toward a pro-inflammatory phenotype. During dextran sodium sulfate (DSS)-induced chronic colitis, the secretion of CSF was augmented by proinflammatory IL-1β, contributing to lowered pain threshold.^103^

The pathogenesis of IBD is accompanied by neurological changes including altered ENS surface marker expression. In humans, increases in the percentage of SP + neurons, NOS + neurons, but not VIP + neurons, have been reported for the myenteric plexus,^173,174^ whilst only VIP + neurons were increased in the submucosal plexus.^174^ Enteric neurons also undergo functional changes during IBD. In guinea-pigs subjected to TNBS-induced colitis, ISNs become hyperexcitable, whilst other neurons exhibit enhanced excitatory synaptic plasticity.^175^ Glial cells also modulate inflammation with the disruption or ablation of enteric glial cells in mice leading to jejuno-ileitis.^77,78,176^ This corresponds with observations that patients with Crohn’s disease exhibit reduced glial cell numbers,^176^ and that treatment with glial-derived s-nitrosoglutathione can restore mucosal barrier function in tissue biopsies from these patient (although the exact nature of these changes remains controversial).^78^

Neuronal loss has also been noted for both the myenteric plexus and submucosal plexus in animal models of trinitrobenzene sulfonic acid (TNBS)-induced IBD.^177,178^ Similarly, myenteric neuronal loss accompanied by dysfunctions of motility is a feature of the GI inflammation seen in the Winnie mouse, a genetic model of IBD in which the gene encoding the muc2 mucin protein is mutated leading to severe inflammation in early adulthood.^179^ By contrast studies in humans or DSS-induced colitis in mice, reported either decreases,^180^ increases^84,181,182^ or no change^174^ in myenteric neuronal numbers. In line with this, one study using TNBS- and DSS-induced colitis models in mice suggested that overall neuronal density is positively associated with the severity of inflammation and may contribute to disease progression.^183^ Thus different studies report disparate influences of IBD on neuronal densities in the myenteric plexus. Why these discrepancies arise, and what they mean, will require further insight into the mechanisms leading to neuronal death or neurogenesis in diseased tissues. During experimental colitis, increased ATP levels were reported to cause neuronal death by activation of neuronal P2X7 receptors.^180^ In the Winnie mouse model neuronal loss was diminished by treatments that reduce oxidative stress.^179^ By contrast, DSS-induced colitis can promote enteric neurogenesis via a 5-HT receptor 4-dependent pathway,^182^ or through the differentiation of Sox2 + glial cells.^84^ Higher numbers of enteric neurons are also observed in humans suffering ulcerative colitis or *C.difficile*-associated colitis, and these neurons express Sox2 indicating neurogenesis from glial cells.^84^

As described above, the reported alterations in enteric neuronal numbers in IBD reveal large discrepancies—both across independent studies and within each disease type. This is likely due to the high heterogeneity of both disorders, and the multifactorial nature of disease contributors which include diet, the microbiota, genetic predisposition, and infectious history. Of note, variability in ENS function across patients might be expected given that IBD can affect different regions of the GI tract. Nevertheless, further studies are warranted, especially with regards to possible **crosstalk** between the ENS and immune cells.

## Infections and the ENS: long-term effects and the recovery of ENS function

Infections can have an impact on the ENS that persists even after the pathogen is cleared. *S. typhimurium* (using the attenuated strain *spiB*) infection leads to neuronal loss in mice lasting up to 4 months,^33^ and *T. spiralis* infection results in attenuated cholinergic signaling and chronically impaired intestinal secretions for up to 2 months following parasite expulsion.^34^ Such long-term changes may also occur for inflammatory diseases, for example animals subjected to TNBS-induced colitis exhibit neuronal loss and increased excitability that persists even after inflammation had subsided.^177,178,184^

Of note long-term alterations to the intestinal function following pathogen infection likely underlies post-infectious (PI-) irritable bowel syndrome (IBS). IBS is generally considered to be an idiopathic physiological syndrome, typically accompanied by low-grade inflammation with a diarrhea-predominant, constipation-predominant or mixed phenotype.^185^ PI-IBS occurs in 10–30% of individuals after acute bacterial, parasitic or viral intestinal infection, mostly displaying a diarrhea-dominant subtype, with symptoms lasting for 6 months and resembling sporadic IBS.^37^ The exact mechanisms driving PI-IBS have yet to be elucidated but disease is often accompanied by inflammation,^186^ microbial dysbiosis,^187^ and alterations to ENS function. Indeed, submucosal neurons from PI-IBS patients are more responsive to capsaicin due to sensitization of a nociceptive receptor, TRPV1, for up to 2 years after disease onset.^188^ Supernatants collected from biopsies of PI-IBS patients activate extrinsic neurons that mediate nociception via TRPV1, a finding that together with altered nerve function may explain the abdominal pain experienced by PI-IBS patients.^188^ Disease is also accompanied by enhanced synaptic sprouting and connectivity of the ENS, likely contributing to intestinal hypercontractility.^189^ Lastly, PI-IBS can be associated with long-term alteration of certain EE cell populations. Analysis from rectal biopsies of PI-IBS patients taken at 6 and 12 weeks after acute infection with Campylobacter enteritis revealed temporary increases in PYY-containing EE cells, but persistent increases in 5-HT containing EC cells (lasting for at least 12 weeks),^190^ both of which would be expected to alter ENS function.

Despite the ability of infection or inflammation to produce long-term changes to the ENS, numerous reports indicate that the ENS has the potential to regenerate and return to a pre-infection or pre-injury state. Neuronal density and morphology recover after resolution of *N. brasiliensis* infection in rats.^191^ But evidence supporting post-infection ENS recovery is limited for other pathogens. The ENS can undergo neurogenesis from glial cells,^29,84,182^ or neural stem cells,^28,31,73^ in response to injury and there is evidence that newly generated neurons can form new synapses within a preformed neuronal network as they express synaptophysin and develop functional varicosities.^29,84^ While neurons differentiating from transplanted neuron precursors have been shown to receive synaptic inputs from the native circuits and form functional synapses with other enteric neurons and with effector tissues.^192,193^ We also know that ENS nerve axons can regenerate following physical damage.^194,195^ In one example, transection and reanastomosis of guinea pig colon was reported to disrupt the peristaltic motility due to lost neuronal innervation and muscle injury. However, by 60 days post-surgery, nerve axons had grown across the re-anastomosis site and peristaltic motility was largely restored.^194^ Thus, multiple pathways can potentially facilitate ENS recovery following pathogen clearance or the resolution of inflammation. In this regard, GDNF promotes axonal outgrowth following TNBS-induced injury, with muscle rather than glial cells reported to be the main source of GDNF.^195^ 5-HT4 receptors also play an important role by limiting apoptosis and promoting neurogenesis.^28,30,182^ Although the exact mechanisms by which this occurs remain unclear, 5-HT4 receptor agonists increase expression of cyclic AMP (cAMP)-responsive element-binding protein^28^ and RET^31,32^ within neurons, both are known to have neurotrophic functions. Importantly, the newly formed neurons and neuronal connections driven by 5-HT4 receptor activation restored proper intestinal functions, including normalizing motility.^30^ Thus manipulating the ENS, for instance via 5-HT4 receptor stimulation, may reduce ENS damage and/or promote ENS recovery. Despite studies focusing on ENS regeneration following infection or inflammation being limited, research in this area could help us understand how the basic biology of the ENS is regulated and hold great potential for development of new approaches to treat intestinal dysfunction and PI-IBS.

## Conclusion and future directions

Given the ENS is involved in mediating every aspect of GI function, we believe its role as an important component of the host protective response should be considered in any investigation of GI infection or inflammation. As outlined in this review, a growing body of literature demonstrates an important role for neuroimmune interactions in regulating both immune cell and neuronal cell function within the GI tract, with extensive communication occurring during homeostasis, infection, and inflammation. In addition, the extrinsic nerves that innervate the GI tract can also communicate with the immune system, while feeding information back to the brain, enabling a systemic response. Identifying new pathways or answering unresolved questions on enteric neuroimmune interactions will undoubtedly advance our understanding of both the immune system and the ENS, as well as providing a solid background for future therapeutic development.

Multiple future research directions need to be pursued. These include the relationship between activity within the enteric neural circuits and the different immune cells under physiological conditions, which mediators are involved and how do they interact to produce the homeostatic mechanisms seen in the healthy GI tract. The obvious further question of what happens to this complex relationship in pathological conditions and how can the healthy interactions be restored. Given that neuroimmune interactions depend on the microbial environment, another major question is how the GI microbiota and its regional distribution affect these interactions.

Although much more is required to understand the complex neuroimmune interactions at the interface between the body and the environment, advances are being made with each of these issues and many others, as highlighted in this review. These recent publications have not only set solid a background for our understanding of the ENS and the immune system but also highlight importance of understanding the complexity of interactions occurring between these two systems. With the continued improvements in methodologies, we can expect an onslaught of exiting discoveries in this field.

## References

[1] Mowat AM, Agace WW (2014). Regional specialization within the intestinal immune system. Nat. Rev. Immunol..

[2] Sender R, Fuchs S, Milo R (2016). Revised Estimates for the Number of Human and Bacteria Cells in the Body. PLOS Biol..

[3] Bornstein, J. C. Autonomic nervous system: gastrointestinal control. (eds Squire L. R.) *Encyc*lopedi*a* o*f Neuroscience*. 929–939 (Academic Press, 2009).

[4] Furness J. B. *The enteric nervous system*. (Blackwell Pub, 2006).

[5] Sperber AD (2020). Worldwide Prevalence and Burden of Functional Gastrointestinal Disorders, Results of Rome Foundation Global Study. Gastroenterology.

[6] Besedovsky H, Sorkin E, Felix D, Haas H (1977). Hypothalamic changes during the immune response. Eur. J. Immunol..

[7] Weihe E (1991). Molecular anatomy of the neuro-immune connection. Int. J. Neurosci..

[8] Yoo BB, Mazmanian SK (2017). The Enteric network: interactions between the immune and nervous systems of the gut. Immunity.

[9] Chesné J, Cardoso V, Veiga-Fernandes H (2019). Neuro-immune regulation of mucosal physiology. Mucosal Immunol..

[10] Veiga-Fernandes H, Mucida D (2016). Neuro-Immune Interactions at Barrier Surfaces. Cell.

[11] Verheijden S, Boeckxstaens GE (2017). Neuroimmune interaction and the regulation of intestinal immune homeostasis. Am. J. Physiol.-Gastrointest. Liver Physiol..

[12] Buhner S, Schemann M (2012). Mast cell–nerve axis with a focus on the human gut. Biochimica et. Biophysica Acta (BBA) - Mol. Basis Dis..

[13] Matteoli G (2014). A distinct vagal anti-inflammatory pathway modulates intestinal muscularis resident macrophages independent of the spleen. Gut.

[14] Muller Paul A (2014). Crosstalk between Muscularis Macrophages and Enteric Neurons Regulates Gastrointestinal Motility. Cell.

[15] Seillet C (2020). The neuropeptide VIP confers anticipatory mucosal immunity by regulating ILC3 activity. Nat. Immunol..

[16] Cardoso V (2017). Neuronal regulation of type 2 innate lymphoid cells via neuromedin U. Nature.

[17] Nagashima H (2019). Neuropeptide CGRP Limits Group 2 Innate Lymphoid Cell Responses and Constrains Type 2 Inflammation. Immunity.

[18] Ibiza S (2016). Glial-cell-derived neuroregulators control type 3 innate lymphoid cells and gut defence. Nature.

[19] Drokhlyansky E (2020). The Human and Mouse Enteric Nervous System at Single-Cell Resolution. Cell.

[20] Jodal M, Wingren U, Jansson M, Heidemann M, Lundgren O (1993). Nerve involvement in fluid transport in the inflamed rat jejunum. Gut.

[21] Argenzio RA, Armstrong M, Rhoads JM (1996). Role of the enteric nervous system in piglet cryptosporidiosis. J. Pharmacol. Exp. Ther..

[22] Moreels TG (2001). Effect of Schistosoma mansoni-induced granulomatous inflammation on murine gastrointestinal motility. Am. J. Physiol.-Gastrointest. Liver Physiol..

[23] Mashaghi A (2016). Neuropeptide substance P and the immune response. Cell. Mol. Life Sci..

[24] Koon HW, Pothoulakis C (2006). Immunomodulatory Properties of Substance P. Ann. N. Y. Acad. Sci..

[25] Chandrasekharan B, Nezami BG, Srinivasan S (2013). Emerging neuropeptide targets in inflammation: NPY and VIP. Am. J. Physiol.-Gastrointest. Liver Physiol..

[26] Gwynne RM, Ellis M, Sjövall H, Bornstein JC (2009). Cholera Toxin Induces Sustained Hyperexcitability in Submucosal Secretomotor Neurons in Guinea Pig Jejunum. Gastroenterology.

[27] Jarret A (2020). Enteric Nervous System-Derived IL-18 Orchestrates Mucosal Barrier Immunity. Cell.

[28] Liu M-T, Kuan Y-H, Wang J, Hen R, Gershon MD (2009). 5-HT4 Receptor-Mediated Neuroprotection and Neurogenesis in the Enteric Nervous System of Adult Mice. J. Neurosci..

[29] Laranjeira C (2011). Glial cells in the mouse enteric nervous system can undergo neurogenesis in response to injury. J. Clin. Investig..

[30] Matsuyoshi H (2010). A 5-HT4-receptor activation-induced neural plasticity enhances in vivo reconstructs of enteric nerve circuit insult. Neurogastroenterol. Motil..

[31] Kawahara I (2012). Comparison of effects of a selective 5-HT reuptake inhibitor versus a 5-HT4 receptor agonist on in vivo neurogenesis at the rectal anastomosis in rats. Am. J. Physiol.-Gastrointest. Liver Physiol..

[32] Goto K, Kawahara I, Kuniyasu H, Takaki M (2015). A protein tyrosine kinase receptor, c-RET signaling pathway contributes to the enteric neurogenesis induced by a 5-HT4 receptor agonist at an anastomosis after transection of the gut in rodents. J. Physiol. Sci..

[33] Matheis F (2020). Adrenergic Signaling in Muscularis Macrophages Limits Infection-Induced Neuronal Loss. Cell.

[34] Venkova K (2006). Greenwood-van Meerveld B. Long-lasting changes in small intestinal transport following the recovery from Trichinella spiralis infection. Neurogastroenterol. Motil..

[35] Barbara G (2009). Postinfectious Irritable Bowel Syndrome. J. Pediatric Gastroenterol. Nutr..

[36] Spiller R, Garsed K (2009). Postinfectious Irritable Bowel Syndrome. Gastroenterology.

[37] Ghoshal UC, Gwee K-A, Post-infectious IBS (2017). tropical sprue and small intestinal bacterial overgrowth: the missing link. Nat. Rev. Gastroenterol. Hepatol..

[38] Jacobson A, Yang D, Vella M, Chiu IM (2021). The intestinal neuro-immune axis: crosstalk between neurons, immune cells, and microbes. Mucosal Immunol..

[39] May-Zhang AA (2021). Combinatorial Transcriptional Profiling of Mouse and Human Enteric Neurons Identifies Shared and Disparate Subtypes In Situ. Gastroenterology.

[40] Morarach K (2021). Diversification of molecularly defined myenteric neuron classes revealed by single-cell RNA sequencing. Nat. Neurosci..

[41] Wright CM (2021). scRNA-Seq Reveals New Enteric Nervous System Roles for GDNF, NRTN, and TBX3. Cell. Mol. Gastroenterol. Hepatol..

[42] Moore BA, Vanner S (2000). Properties of synaptic inputs from myenteric neurons innervating submucosal S neurons in guinea pig ileum. Am. J. Physiol..

[43] Galligan JJ, Surprenant A, Tonini M, North RA (1988). Differential localization of 5-HT_1_ receptors on myenteric and submucosal neurons. Am. J. Physiol..

[44] Kirchgessner AL, Tamir H, Gershon MD (1992). Identification and stimulation by serotonin of intrinsic sensory neurons of the submucosal plexus of the guinea pig gut: activity-induced expression of Fos immunoreactivity. J. Neurosci..

[45] Monro RL, Bornstein JC, Bertrand PP (2008). Synaptic transmission from the submucosal plexus to the myenteric plexus in guinea-pig ileum. Neurogastroenterol. Motil..

[46] Song Z-M, Brookes SJH, Steele PA, Costa M (1992). Projections and pathways of submucous neurons to the mucosa of the guinea-pig small intestine. Cell TissRes.

[47] Li Z (2019). Regional complexity in enteric neuron wiring reflects diversity of motility patterns in the mouse large intestine. eLife.

[48] Foong JPP, Tough IR, Cox HM, Bornstein JC (2014). Properties of cholinergic and non-cholinergic submucosal neurons along the mouse colon. J. Physiol..

[49] Furness JB (2012). The enteric nervous system and neurogastroenterology. Nat. Rev. Gastroenterol. Hepatol..

[50] Bohórquez DV (2015). Neuroepithelial circuit formed by innervation of sensory enteroendocrine cells. J. Clin. Investig..

[51] Jones LA, Sun WE, Martin AM, Keating DJ (2020). The ever-changing roles of serotonin. Int. J. Biochem. Cell Biol..

[52] Gershon MD (2013). 5-Hydroxytryptamine (serotonin) in the gastrointestinal tract. Curr. Opin. Endocrinol. Diabetes Obes..

[53] Bellono NW (2017). Enterochromaffin cells are gut chemosensors that couple to sensory neural pathways. Cell.

[54] Nozawa K (2009). TRPA1 regulates gastrointestinal motility through serotonin release from enterochromaffin cells. Proc. Natl Acad. Sci. USA.

[55] Spencer NJ, Costa M, Hibberd TJ, Wood JD (2021). Advances in colonic motor complexes in mice. Am. J. Physiol. Gastrointest. Liver Physiol..

[56] Heredia DJ (2013). Important role of mucosal serotonin in colonic propulsion and peristaltic reflexes: in vitro analyses in mice lacking tryptophan hydroxylase 1. J. Physiol. (Lond.).

[57] Gwynne RM, Clarke AJ, Furness JB, Bornstein JC (2015). Both exogenous 5-HT and endogenous 5-HT, released by fluoxetine, enhance distension evoked propulsion in guinea-pig ileum in vitroBoth exogenous 5-HT and endogenous 5-HT, released by fluoxetine, enhance distension evoked propulsion in guinea-pig ileum in vitro. Front. Neurosci..

[58] Ellis M, Chambers JD, Gwynne RM, Bornstein JC (2013). Serotonin (5-HT) and cholecystokinin (CCK) mediate nutrient induced segmentation in guinea pig small intestine. Am. J. Physiol. Gastrointest. Liver Physiol..

[59] Balasuriya GK, Hill-Yardin EL, Gershon MD, Bornstein JC (2016). A sexually dimorphic effect of cholera toxin: rapid changes in colonic motility mediated via a 5-HT3 receptor-dependent pathway in female C57Bl/6 mice. J. Physiol..

[60] Bertrand PP (2006). Real-time measurement of serotonin release and motility in guinea pig ileum. J. Physiol. (Lond.).

[61] Lundgren O (2002). Enteric nerves and diarrhoea. Pharm. Toxicol..

[62] Qu ZD (2008). Immunohistochemical analysis of neuron types in the mouse small intestine. Cell Tissue Res..

[63] Costa M (1996). Neurochemical classification of myenteric neurons in the guinea-pig ileum. Neuroscience.

[64] Bornstein JC, Costa M, Grider JR (2004). Enteric motor and interneuronal circuits controlling motility. Neurogastroenterol. Motil..

[65] Gwynne RM, Bornstein JC (2007). Synaptic transmission at functionally identified synapses in the enteric nervous system: roles for both ionotropic and metabotropic receptors. Curr. Neuropharmacol..

[66] Bornstein, J. C. & Foong J. P. P. Enteric neural regulation of mucosal secretion. Vol. 1. (eds Said H. M.). *Physiology of the Gastrointestinal Tract*, 6th edn. 429–451 (Academic Press, Elsevier, 2018).

[67] Furness JB, Costa M, Keast JR (1984). Choline acetyltransferase and peptide immunoreactivity of submucous neurons in the small intestine of the guinea-pig. Cell Tissue Res..

[68] Evangelista S (2014). Capsaicin receptor as target of calcitonin gene-related peptide in the gut. Prog. Drug Res..

[69] Vanden Berghe P, Klingauf J (2007). Spatial organization and dynamic properties of neurotransmitter release sites in the enteric nervous system. Neuroscience.

[70] Brock JA, Cunnane TC (1988). Electrical activity at the sympathetic neuroeffector junction in the guinea-pig vas deferens. J. Physiol. (Lond.).

[71] Pham TD, Gershon MD, Rothman TP (1991). Time of origin of neurons in the murine enteric nervous system: sequence in relation to phenotype. J. Comp. Neurol..

[72] Gianino S, Grider JR, Cresswell J, Enomoto H, Heuckeroth RO (2003). GDNF availability determines enteric neuron number by controlling precursor proliferation. Development.

[73] Kulkarni S (2017). Adult enteric nervous system in health is maintained by a dynamic balance between neuronal apoptosis and neurogenesis. Proc. Natl Acad. Sci..

[74] Spencer NJ, Hu H (2020). Enteric nervous system: sensory transduction, neural circuits and gastrointestinal motility. Nat. Rev. Gastroenterol. Hepatol..

[75] Gulbransen BD, Sharkey KA (2009). Purinergic neuron-to-glia signaling in the enteric nervous system. Gastroenterology.

[76] Grubišić V, Verkhratsky A, Zorec R, Parpura V (2018). Enteric glia regulate gut motility in health and disease. Brain Res. Bull..

[77] Bush TG (1998). Fulminant Jejuno-Ileitis following Ablation of Enteric Glia in Adult Transgenic Mice. Cell.

[78] Savidge TC (2007). Enteric Glia Regulate Intestinal Barrier Function and Inflammation Via Release of S-Nitrosoglutathione. Gastroenterology.

[79] Hanani M (2000). Patch-clamp study of neurons and glial cells in isolated myenteric ganglia. Am. J. Physiol..

[80] Boesmans W (2019). Structurally defined signaling in neuro-glia units in the enteric nervous system. Glia.

[81] Fung C (2017). VPAC receptor subtypes tune purinergic neuron-to-glia communication in the murine submucosal plexus. Front. Cell. Neurosci..

[82] Delvalle NM, Fried DE, Rivera-Lopez G, Gaudette L, Gulbransen BD (2018). Cholinergic activation of enteric glia is a physiological mechanism that contributes to the regulation of gastrointestinal motility. Am. J. Physiol. Gastrointest. Liver Physiol..

[83] Gulbransen BD, Sharkey KA (2012). Novel functional roles for enteric glia in the gastrointestinal tract. Nat. Rev. Gastroenterol. Hepatol..

[84] Belkind-Gerson J (2017). Colitis promotes neuronal differentiation of Sox2+ and PLP1+ enteric cells. Sci. Rep..

[85] Chow AK, Gulbransen BD (2016). Potential roles of enteric glia in bridging neuroimmune communication in the gut. Am. J. Physiol.-Gastrointest. Liver Physiol..

[86] Veiga-Fernandes H, Pachnis V (2017). Neuroimmune regulation during intestinal development and homeostasis. Nat. Immunol..

[87] Seguella L, Gulbransen BD (2021). Enteric glial biology, intercellular signalling and roles in gastrointestinal disease. Nat. Rev. Gastroenterol. Hepatol..

[88] Chistiakov DA, Bobryshev YV, Kozarov E, Sobenin IA, Orekhov AN (2015). Intestinal mucosal tolerance and impact of gut microbiota to mucosal tolerance. Front. Microbiol..

[89] Perez-Lopez A, Behnsen J, Nuccio S-P, Raffatellu M (2016). Mucosal immunity to pathogenic intestinal bacteria. Nat. Rev. Immunol..

[90] Harris NL, Pn Loke (2017). Recent Advances in Type-2-Cell-Mediated. Immun.: Insights Helminth Infect. Immun..

[91] Margolis KG, Gershon MD, Bogunovic M (2016). Cellular Organization of Neuroimmune Interactions in the Gastrointestinal Tract. Trends Immunol..

[92] Yan Y (2021). Interleukin-6 produced by enteric neurons regulates the number and phenotype of microbe-responsive regulatory T cells in the gut. Immunity.

[93] Talbot J (2020). neuron–ILC3 circuit regulates the intestinal barrier. Nature.

[94] Sang Q, Young HM (1998). The identification and chemical coding of cholinergic neurons in the small and large intestine of the mouse. Anat. Rec..

[95] Sang Q, Young HM (1996). Chemical coding of neurons in the myenteric plexus and external muscle of the small and large intestine of the mouse. Cell Tissue Res..

[96] Mongardi Fantaguzzi C, Thacker M, Chiocchetti R, Furness JB (2009). Identification of neuron types in the submucosal ganglia of the mouse ileum. Cell Tissue Res..

[97] Goverse G, Stakenborg M, Matteoli G (2016). The intestinal cholinergic anti-inflammatory pathway. J. Physiol..

[98] Ren C, Tong Y-L, Li J-C, Lu Z-Q, Yao Y-M (2017). The Protective Effect of Alpha 7 Nicotinic Acetylcholine Receptor Activation on Critical Illness and Its Mechanism. Int. J. Biol. Sci..

[99] Herr N, Bode C, Duerschmied D (2017). The Effects of Serotonin in Immune Cells. Front. Cardiovasc. Med..

[100] Donato R (2009). S100B’s double life: Intracellular regulator and extracellular signal. Biochimica et. Biophysica Acta (BBA) - Mol. Cell Res..

[101] Kunze WAA, Furness JB (1999). The enteric nervous system and regulation of intestinal motility. Annu. Rev. Physiol..

[102] Bharucha, A. E. & Hasler, W. L. Motility of the Small Intestine and Colon. *Yamada’s Textbook of Gastroenterology*. 367–385 (John Wiley & Sons, UK, 2015).

[103] Grubišić V (2020). Enteric Glia Modulate Macrophage Phenotype and Visceral Sensitivity following Inflammation. Cell Rep..

[104] The FO (2007). Activation of the Cholinergic Anti-Inflammatory Pathway Ameliorates Postoperative Ileus in Mice. Gastroenterology.

[105] Becker L (2018). Age-dependent shift in macrophage polarisation causes inflammation-mediated degeneration of enteric nervous system. Gut.

[106] Saffrey MJ (2013). Cellular changes in the enteric nervous system during ageing. Dev. Biol..

[107] Li S, Qi D, Li J-N, Deng X-Y, Wang D-X (2021). Vagus nerve stimulation enhances the cholinergic anti-inflammatory pathway to reduce lung injury in acute respiratory distress syndrome via STAT3. Cell Death Discov..

[108] Chen Z (2021). Interleukin-33 Promotes Serotonin Release from Enterochromaffin Cells for Intestinal Homeostasis. Immunity.

[109] Cooke HJ, Sidhu M, Wang YZ (1997). 5-HT activates neural reflexes regulating secretion in the guinea-pig colon. Neurogastroenterol. Motil..

[110] Molofsky Ari B, Savage Adam K, Locksley, Richard M (2015). Interleukin-33 in Tissue Homeostasis, Injury, and Inflammation. Immunity.

[111] Kaelberer MM (2018). A gut-brain neural circuit for nutrient sensory transduction. Science.

[112] Collins J, Borojevic R, Verdu EF, Huizinga JD, Ratcliffe EM (2014). Intestinal microbiota influence the early postnatal development of the enteric nervous system. Neurogastroenterol. Motil..

[113] McVey Neufeld KA, Mao YK, Bienenstock J, Foster JA, Kunze WA (2013). The microbiome is essential for normal gut intrinsic primary afferent neuron excitability in the mouse. Neurogastroenterol. Motil..

[114] Hung LY (2019). Neonatal Antibiotics Disrupt Motility and Enteric Neural Circuits in Mouse Colon. Cell. Mol. Gastroenterol. Hepatol..

[115] Obata Y (2020). Neuronal programming by microbiota regulates intestinal physiology. Nature.

[116] Grasa L (2015). Antibiotic-Induced Depletion of Murine Microbiota Induces Mild Inflammation and Changes in Toll-Like Receptor Patterns and Intestinal Motility. Microb. Ecol..

[117] Caputi V (2017). Antibiotic-induced dysbiosis of the microbiota impairs gut neuromuscular function in juvenile mice. Br. J. Pharm..

[118] Kabouridis Panagiotis S (2015). Microbiota Controls the Homeostasis of Glial Cells in the Gut Lamina Propria. Neuron.

[119] Barajon I (2009). Toll-like Receptors 3, 4, and 7 Are Expressed in the Enteric Nervous System and Dorsal Root Ganglia. J. Histochem. Cytochem..

[120] Brun P (2013). Toll-Like Receptor 2 Regulates Intestinal Inflammation by Controlling Integrity of the Enteric Nervous System. Gastroenterology.

[121] Esposito G (2014). Palmitoylethanolamide improves colon inflammation through an enteric glia/toll like receptor 4-dependent PPAR-α activation. Gut.

[122] McVey Neufeld KA, Perez-Burgos A, Mao YK, Bienenstock J, Kunze WA (2015). The gut microbiome restores intrinsic and extrinsic nerve function in germ-free mice accompanied by changes in calbindin. Neurogastroenterol. Motil..

[123] De Vadder F (2018). Gut microbiota regulates maturation of the adult enteric nervous system via enteric serotonin networks. Proc. Natl Acad. Sci..

[124] Neuvonen MI (2018). Intestinal Microbiota in Hirschsprung Disease. J. Pediatric Gastroenterol. Nutr..

[125] Rolig AS (2017). The enteric nervous system promotes intestinal health by constraining microbiota composition. PLOS Biol..

[126] Hosie S (2019). Gastrointestinal dysfunction in patients and mice expressing the autism-associated R451C mutation in neuroligin-3. Autism Res..

[127] Klimovich A (2020). Prototypical pacemaker neurons interact with the resident microbiota. Proc. Natl Acad. Sci..

[128] Murillo-Rincon AP (2017). Spontaneous body contractions are modulated by the microbiome of Hydra. Sci. Rep..

[129] Augustin R (2017). A secreted antibacterial neuropeptide shapes the microbiome of Hydra. Nat. Commun..

[130] Haan LD, Hirst TR (2004). Cholera toxin: a paradigm for multi-functional engagement of cellular mechanisms (Review). Mol. Membr. Biol..

[131] Koussoulas K, Gwynne RM, Foong JPP, Bornstein JC (2017). Cholera Toxin Induces Sustained Hyperexcitability in Myenteric, but Not Submucosal, AH Neurons in Guinea Pig Jejunum. Front. Physiol..

[132] Fung C (2018). Cholinergic Submucosal Neurons Display Increased Excitability Following in Vivo Cholera Toxin Exposure in Mouse Ileum. Front. Physiol..

[133] Kordasti S (2006). Effects of cholera toxin on the potential difference and motor responses induced by distension in the rat proximal small intestine in vivo. Am. J. Physiol. Gastrointest. Liver Physiol..

[134] Xia Y, Hu HZ, Liu S, Pothoulakis C, Wood JD (2000). *Clostridium difficile* toxin A excites enteric neurones and suppresses sympathetic neurotransmission in the guinea pig. Gut.

[135] Mantyh CR (1996). Substance P activation of enteric neurons in response to intraluminal Clostridium difficile toxin A in the rat ileum. Gastroenterology.

[136] Flegel WA (1991). Cytokine response by human monocytes to Clostridium difficile toxin A and toxin B. Infect. Immun..

[137] Neunlist M (2003). Toxin B of Clostridium difficile activates human VIP submucosal neurons, in part via an IL-1β-dependent pathway. Am. J. Physiol.-Gastrointest. Liver Physiol..

[138] Tixier E, Lalanne F, Just I, Galmiche J-P, Neunlist M (2005). Human mucosa/submucosa interactions during intestinal inflammation: involvement of the enteric nervous system in interleukin-8 secretion. Cell. Microbiol..

[139] Stebbing MJ, Bornstein JC (1996). Electrophysiological mapping of fast excitatory synaptic inputs to morphologically and chemically characterized myenteric neurons of guinea-pig small intestine. Neuroscience.

[140] El Karim IA, Linden GJ, Orr DF, Lundy FT (2008). Antimicrobial activity of neuropeptides against a range of micro-organisms from skin, oral, respiratory and gastrointestinal tract sites. J. Neuroimmunol..

[141] Campos-Salinas J (2014). Therapeutic Efficacy of Stable Analogues of Vasoactive Intestinal Peptide against Pathogens. J. Biol. Chem..

[142] Allen JE, Maizels RM (2011). Diversity and dialogue in immunity to helminths. Nat. Rev. Immunol..

[143] von Moltke J, Ji M, Liang H-E, Locksley RM (2016). Tuft-cell-derived IL-25 regulates an intestinal ILC2–epithelial response circuit. Nature.

[144] Finkelman FD (2004). Interleukin-4- and interleukin-13-mediated host protection against intestinal nematode parasites. Immunological Rev..

[145] Halliez MCM, Buret AG (2015). Gastrointestinal Parasites and the Neural Control of Gut Functions. Front. Cell. Neurosci..

[146] Palmer JM, Wong-Riley M, Sharkey KA (1998). Functional alterations in jejunal myenteric neurons during inflammation in nematode-infected guinea pigs. Am. J. Physiol.-Gastrointest. Liver Physiol..

[147] Zhao A (2003). Dependence of IL-4, IL-13, and Nematode-Induced Alterations in Murine Small Intestinal Smooth Muscle Contractility on Stat6 and Enteric Nerves. J. Immunol..

[148] Dwinell KL, Bass P, Zou F, Oaks JA (2002). Small intestinal transections decrease the occurrence of tapeworm-induced myoelectric patterns in the rat. Neurogastroenterol. Motil..

[149] McKay DM, Fairweather I (1997). A role for the enteric nervous system in the response to helminth infections. Parasitol. Today.

[150] Balemba OB, Semuguruka WD, Hay-Schmidt A, Johansen MV, Dantzer V (2001). Vasoactive intestinal peptide and substance P-like immunoreactivities in the enteric nervous system of the pig correlate with the severity of pathological changes induced by Schistosoma japonicum. Int J. Parasitol..

[151] Collins SM, Blennerhassett PA, Blennerhassett MG, Vermillion DL (1989). Impaired acetylcholine release from the myenteric plexus of Trichinella-infected rats. Am. J. Physiol.-Gastrointest. Liver Physiol..

[152] Garza A, Weinstock J, Robinson P (2008). Absence of the SP/SP Receptor Circuitry in the Substance P-Precursor Knockout Mice or SP Receptor, Neurokinin (NK)1 Knockout Mice Leads to an Inhibited Cytokine Response in Granulomas Associated with Murine Taenia crassiceps Infection. J. Parasitol..

[153] Nussbaum JC (2013). Type 2 innate lymphoid cells control eosinophil homeostasis. Nature.

[154] Xu H (2019). Transcriptional Atlas of Intestinal Immune Cells Reveals that Neuropeptide α-CGRP Modulates Group 2 Innate Lymphoid Cell Responses. Immunity.

[155] Klose CSN (2017). The neuropeptide neuromedin U stimulates innate lymphoid cells and type 2 inflammation. Nature.

[156] Furness JB, Pompolo S, Murphy R, Giraud A (1989). Projections of neurons with neuromedin U-like immunoreactivity in the small intestine of the guinea-pig. Cell Tissue Res..

[157] McCoy ES, Taylor-Blake B, Zylka MJ (2012). CGRPα-Expressing Sensory Neurons Respond to Stimuli that Evoke Sensations of Pain and Itch. PLOS ONE.

[158] Chu C (2021). The ChAT-acetylcholine pathway promotes group 2 innate lymphoid cell responses and anti-helminth immunity. Sci. Immunol..

[159] Roberts LB (2021). Acetylcholine production by group 2 innate lymphoid cells promotes mucosal immunity to helminths. Sci. Immunol..

[160] Buhner S (2017). Calcium Imaging of Nerve-Mast Cell Signaling in the Human Intestine. Front. Physiol..

[161] Wood JD (2004). Enteric neuroimmunophysiology and pathophysiology1,2. Gastroenterology.

[162] Kreider T, Anthony RM, Urban JF, Gause WC (2007). Alternatively activated macrophages in helminth infections. Curr. Opin. Immunol..

[163] Wang F (2021). A basophil-neuronal axis promotes itch. Cell.

[164] Henry EK, Inclan-Rico JM, Siracusa MC (2017). Type 2 Cytokine Responses: regulating Immunity to Helminth Parasites and Allergic Inflammation. Curr. Pharmacol. Rep..

[165] Crawford SE (2017). Rotavirus infection. Nat. Rev. Dis. Prim..

[166] Hellysaz A, Hagbom M (2021). Understanding the Central Nervous System Symptoms of Rotavirus: a qualitative review. Viruses.

[167] White JP (2018). Intestinal Dysmotility Syndromes following Systemic Infection by Flaviviruses. Cell.

[168] Desai P (2021). Enteric helminth coinfection enhances host susceptibility to neurotropic flaviviruses via a tuft cell-IL-4 receptor signaling axis. Cell.

[169] Strober W, Fuss IJ (2011). Proinflammatory Cytokines in the Pathogenesis of Inflammatory Bowel Diseases. Gastroenterology.

[170] Steinbach EC, Plevy SE (2013). The Role of Macrophages and Dendritic Cells in the Initiation of Inflammation in IBD. Inflamm. Bowel Dis..

[171] Abraham C, Cho JH (2009). Inflammatory Bowel Disease. N. Engl. J. Med..

[172] Xia Y (1999). IL-1β and IL-6 excite neurons and suppress nicotinic and noradrenergic neurotransmission in guinea pig enteric nervous system. J. Clin. Investig..

[173] Neunlist M (2003). Changes in chemical coding of myenteric neurones in ulcerative colitis. Gut.

[174] Boyer L, Sidpra D, Jevon G, Buchan AM, Jacobson K (2007). Differential responses of VIPergic and nitrergic neurons in paediatric patients with Crohn’s disease. Autonomic Neurosci..

[175] Lomax AE, Mawe GM, Sharkey KA (2005). Synaptic facilitation and enhanced neuronal excitability in the submucosal plexus during experimental colitis in guinea-pig. J. Physiol..

[176] Cornet A (2001). Enterocolitis induced by autoimmune targeting of enteric glial cells: a possible mechanism in Crohn’s disease?. Proc. Natl Acad. Sci..

[177] Linden DR (2005). Indiscriminate loss of myenteric neurones in the TNBS-inflamed guinea-pig distal colon. Neurogastroenterol. Motil..

[178] Sanovic S, Lamb DP, Blennerhassett MG (1999). Damage to the Enteric Nervous System in Experimental Colitis. Am. J. Pathol..

[179] Sahakian L (2021). Inhibition of APE1/Ref-1 Redox Signaling Alleviates Intestinal Dysfunction and Damage to Myenteric Neurons in a Mouse Model of Spontaneous Chronic Colitis. Inflamm. Bowel Dis..

[180] Gulbransen BD (2012). Activation of neuronal P2X7 receptor–pannexin-1 mediates death of enteric neurons during colitis. Nat. Med..

[181] Villanacci V (2008). Enteric nervous system abnormalities in inflammatory bowel diseases. Neurogastroenterol. Motil..

[182] Belkind-Gerson J (2015). Colitis Induces Enteric Neurogenesis Through a 5-HT4–dependent Mechanism. Inflamm. Bowel Dis..

[183] Margolis KG (2011). Enteric Neuronal Density Contributes to the Severity of Intestinal Inflammation. Gastroenterology.

[184] Lomax AE, O’Hara JR, Hyland NP, Mawe GM, Sharkey KA (2007). Persistent alterations to enteric neural signaling in the guinea pig colon following the resolution of colitis. Am. J. Physiol.-Gastrointest. Liver Physiol..

[185] Wood, J. D. Chapter 67 - Neuropathophysiology of the Irritable Bowel Syndrome. (eds Said H. M.). *Physiology of the Gastrointestinal Tract* (*Sixth Edition*). 1643–1668 (Academic Press, 2018).

[186] Gwee K-A (2003). Increased rectal mucosal expression of interleukin 1β in recently acquired post-infectious irritable bowel syndrome. Gut.

[187] Jalanka-Tuovinen J (2014). Faecal microbiota composition and host–microbe cross-talk following gastroenteritis and in postinfectious irritable bowel syndrome. Gut.

[188] Balemans D (2017). Evidence for long-term sensitization of the bowel in patients with post-infectious-IBS. Sci. Rep..

[189] Zhang L (2019). EphrinB2/ephB2-mediated myenteric synaptic plasticity: mechanisms underlying the persistent muscle hypercontractility and pain in postinfectious IBS. FASEB J..

[190] Spiller RC (2000). Increased rectal mucosal enteroendocrine cells, T lymphocytes, and increased gut permeability following acute *Campylobacter* enteritis and in post-dysenteric irritable bowel syndrome. Gut.

[191] Stead R, Kosecka-Janiszewska U, Oestreicher A, Dixon M, Bienenstock J (1991). Remodeling of B-50 (GAP-43)- and NSE-immunoreactive mucosal nerves in the intestines of rats infected with Nippostrongylus brasiliensis. J. Neurosci..

[192] Hotta R (2013). Transplanted progenitors generate functional enteric neurons in the postnatal colon. J. Clin. Investig..

[193] Stamp LA (2017). Optogenetic Demonstration of Functional Innervation of Mouse Colon by Neurons Derived From Transplanted Neural Cells. Gastroenterology.

[194] Galligan JJ, Furness JB, Costa M (1989). Migration of the myoelectric complex after interruption of the myenteric plexus: Intestinal transection and regeneration of enteric nerves in the guinea pig. Gastroenterology.

[195] Gougeon PY (2013). The pro-inflammatory cytokines IL-1β and TNFα are neurotrophic for enteric neurons. J. Neurosci..

