# Correction to: Enteric neuroimmune interactions coordinate intestinal responses in health and disease

**DOI:** 10.1038/s41385-021-00459-7

**Published:** 2021-09-30

**Authors:** Haozhe Wang, Jaime P. P. Foong, Nicola L. Harris, Joel C. Bornstein

**Affiliations:** 1 Department of Immunology and Pathology, Central Clinical School, Monash University, The Alfred Centre, Melbourne, VIC Australia; 2 Department of Anatomy and Physiology, University of Melbourne, Melbourne, VIC Australia

Correction to: *Mucosal Immunology* 10.1038/s41385-021-00443-1, published online 1 September 2021

The original version of this article unfortunately was missing requisite credit lines in Figs. 1–4. Images presented in Fig. 1 adapted from Foong JPP, Hung LY, Poon S, Savidge TC, Bornstein JC. Early life interaction between the microbiota and the enteric nervous system. American Journal of Physiology-Gastrointestinal and Liver Physiology 2020;319(5):G541–G548, and modified by the authors. Images presented in Figs. 2–4 adapted from Servier Medical Art (http://smart.servier.com/) and modified by the authors under the following terms: Creative Commons Attribution 3.0 Unported (CC BY 3.0). The original article has been corrected.

